# Changing how we evaluate research is difficult, but not impossible

**DOI:** 10.7554/eLife.58654

**Published:** 2020-08-12

**Authors:** Anna Hatch, Stephen Curry

**Affiliations:** 1 DORARockvilleUnited States; 2 Imperial CollegeLondonUnited Kingdom

**Keywords:** research assessment, research culture, culture change, institutional change, careers in science, None

## Abstract

The San Francisco Declaration on Research Assessment (DORA) was published in 2013 and described how funding agencies, institutions, publishers, organizations that supply metrics, and individual researchers could better evaluate the outputs of scientific research. Since then DORA has evolved into an active initiative that gives practical advice to institutions on new ways to assess and evaluate research. This article outlines a framework for driving institutional change that was developed at a meeting convened by DORA and the Howard Hughes Medical Institute. The framework has four broad goals: understanding the obstacles to changes in the way research is assessed; experimenting with different approaches; creating a shared vision when revising existing policies and practices; and communicating that vision on campus and beyond.

## Introduction

Declarations can inspire revolutionary change, but the high ideals inspiring the revolution must be harnessed to clear guidance and tangible goals to drive effective reform. When the San Francisco Declaration on Research Assessment (DORA) was published in 2013, it catalogued the problems caused by the use of journal-based indicators to evaluate the performance of individual researchers, and provided 18 recommendations to improve such evaluations. Since then, DORA has inspired many in the academic community to challenge long-standing research assessment practices, and over 150 universities and research institutions have signed the declaration and committed to reform.

But experience has taught us that this is not enough to change how research is assessed. Given the scale and complexity of the task, additional measures are called for. We have to support institutions in developing the processes and resources needed to implement responsible research assessment practices. That is why DORA has transformed itself from a website collecting signatures to a broader campaigning initiative that can provide practical guidance. This will help institutions to seize the opportunities created by the momentum now building across the research community to reshape how we evaluate research.

Systemic change requires fundamental shifts in policies, processes and power structures, as well as in deeply held norms and values. Those hoping to drive such change need to understand all the stakeholders in the system: in particular, how do they interact with and depend on each other, and how do they respond to internal and external pressures? To this end DORA and the Howard Hughes Medical Institute (HHMI) convened a meeting in October 2019 that brought together researchers, university administrators, librarians, funders, scientific societies, non-profits and other stakeholders to discuss these questions. Those taking part in the meeting (https://sfdora.org/assessingresearch/agenda/) discussed emerging policies and practices in research assessment, and how they could be aligned with the academic missions of different institutions.

The discussion helped to identify what institutional change could look like, to surface new ideas, and to formulate practical guidance for research institutions looking to embrace reform. This guidance – summarized below – provides a framework for action that consists of four broad goals: i) understand obstacles that prevent change; ii) experiment with different ideas and approaches at all levels; iii) create a shared vision for research assessment when reviewing and revising policies and practices; iv) communicate that vision on campus and externally to other research institutions.

## Understand obstacles that prevent change

Most academic reward systems rely on proxy measures of quality to assess researchers. This is problematic when there is an over-reliance on these proxy measures, particularly so if aggregate measures are used that mask the variations between individuals and individual outputs. Journal-based metrics and the H-index, alongside qualitative notions of publisher prestige and institutional reputation, present obstacles to change that have become deeply entrenched in academic evaluation. This has happened because such measures contain an appealing kernel of meaning (though the appeal only holds so long as one operates within the confines of the law of averages) and because they provide a convenient shortcut for busy evaluators. Additionally, the over-reliance on proxy measures that tend to be focused on research can discourage researchers from working on other activities that are also important to the mission of most research institutions, such as teaching, mentoring, and work that has societal impact.

Rethinking research assessment therefore means addressing the privilege that exists in academia, and taking proper account of how luck and opportunity can influence decision-making more than personal characteristics such as talent, skill and tenacity.

The use of proxy measures also preserves biases against scholars who still feel the force of historical and geographical exclusion from the research community. Progress toward gender and race equality has been made in recent years, but the pace of change remains unacceptably slow. A recent study of basic science departments in US medical schools suggests that under current practices, a level of faculty diversity representative of the national population will not be achieved until 2080 (Gibbs et al., 2016).

Rethinking research assessment therefore means addressing the privilege that exists in academia, and taking proper account of how luck and opportunity can influence decision-making more than personal characteristics such as talent, skill and tenacity. As a community, we need to take a hard look – without averting our gaze from the prejudices that attend questions of race, gender, sexuality, or disability – at what we really mean when we talk about 'success' and 'excellence' if we are to find answers congruent with our highest aspirations.

This is by no means easy. Many external and internal pressures stand in the way of meaningful change. For example, institutions have to wrestle with university rankings as part of research assessment reform, because stepping away from the surrogate, selective, and incomplete 'measures' of performance totted up by rankers poses a reputational threat. Grant funding, which is commonly seen as an essential signal of researcher success, is clearly crucial for many universities and research institutions: however, an overemphasis on grants in decisions about hiring, promotion and tenure incentivizes researchers to discount other important parts of their job. The huge mental health burden of hyper-competition is also a problem that can no longer be ignored (Wellcome, 2020a).

## Experiment with different ideas and approaches at all levels

Culture change is often driven by the collective force of individual actions. These actions take many forms, but spring from a common desire to champion responsible research assessment practices. At the DORA/HHMI meeting Needhi Bhalla (University of California, Santa Cruz) advocated strategies that have been proven to increase equity in faculty hiring – including the use of diversity statements to assess whether a candidate is aligned with the department's equity mission – as part of a more holistic approach to researcher evaluation (Bhalla, 2019). She also described how broadening the scope of desirable research interests in the job descriptions for faculty positions in chemistry at the University of Michigan resulted in a two-fold increase of applicants from underrepresented groups (Stewart and Valian, 2018). As a further step, Bhalla's department now includes untenured assistant professors in tenure decisions: this provides such faculty with insights into the tenure process.

The seeds planted by individual action must be encouraged to grow, so that discussions about research assessment can reach across the entire institution.

The actions of individual researchers, however exemplary, are dependent on career stage and position: commonly, those with more authority have more influence. As chair of the cell biology department at the University of Texas Southwestern Medical Center, Sandra Schmid used her position to revise their hiring procedure to focus on key research contributions, rather than publication or grant metrics, and to explore how the applicant's future plans might best be supported by the department. According to Schmid, the department's job searches were given real breadth and depth by the use of Skype interviews (which enhanced the shortlisting process by allowing more candidates to be interviewed) and by designating faculty advocates from across the department for each candidate (Schmid, 2017). Another proposal for shifting the attention of evaluators from proxies to the content of an applicant's papers and other contributions is to instruct applicants for grants and jobs to remove journal names from CVs and publication lists (Lobet, 2020).

The seeds planted by individual action must be encouraged to grow, so that discussions about research assessment can reach across the entire institution. This is rarely straightforward, given the size and organizational autonomy within modern universities, which is why some have set up working groups to review their research assessment policies and practices. At the Universitat Oberta de Catalunya (UOC) and Imperial College London, for example, the working groups produced action plans or recommendations that have been adopted by the university and are now being implemented (UOC, 2019; Imperial College, 2020). University Medical Center (UMC) Utrecht has gone a step further: in addition to revising its processes and criteria for promotion and for internal evaluation of research programmes (Benedictus et al., 2016), it is undertaking an in-depth evaluation of how the changes are impacting their researchers (see below).

To increase their chances of success these working groups need to ensure that women and other historically excluded groups have a voice. It is also important that the viewpoints of administrators, librarians, tenured and non-tenured faculty members, postdocs, and graduate students are all heard. This level of inclusion is important because when communities impacted by new practices are involved in their design, they are more likely to adopt them. But the more views there are around the table, the more difficult it can be to reach a consensus. Everyone brings their own frame-of-reference, their own ideas, and their own experiences. To help ensure that working groups do not become mired in minutiae, their objectives should be defined early in the process and should be simple, clear and realistic.

## Create a shared vision

### Aligning policies and practices with an institution's mission

The re-examination of an institution's policies and procedures can reveal the real priorities that may be glossed over in aspirational mission statements. Although the journal impact factor (JIF) is widely discredited as a tool for research assessment, more than 40% of research-intensive universities in the United States and Canada explicitly mention the JIF in review, promotion, and tenure documents (McKiernan et al., 2019). The number of institutions where the JIF is not mentioned in such documents, but is understood informally to be a performance criterion, is not known. A key task for working groups is therefore to review how well the institution's values, as expressed in its mission statement, are embedded in its hiring, promotion, and tenure practices. Diversity, equity, and inclusion are increasingly advertised as core values, but work in these areas is still often lumped into the service category, which is the least recognized type of academic contribution when it comes to promotion and tenure (Schimanski and Alperin, 2018).

A complicating factor here is that while mission statements publicly signal organizational values, the commitments entailed by those statements are delivered by individuals, who are prone to unacknowledged biases, such as the perception gap between what people say they value and what they think others hold most dear. For example, when Meredith Niles and colleagues surveyed faculty at 55 institutions, they found that academics value readership most when selecting where to publish their work (Niles et al., 2019). But when asked how their peers decide to publish, a disconnect was revealed: most faculty members believe their colleagues make choices based on the prestige of the journal or publisher. Similar perception gaps are likely to be found when other performance proxies (such as grant funding and student satisfaction) are considered.

Bridging perception gaps requires courage and honesty within any institution – to break with the metrics game and create evaluation processes that are visibly infused with the organization's core values. To give one example, HHMI tries to advance basic biomedical research for the benefit of humanity by setting evaluation criteria that are focused on quality and impact. To increase transparency, these criteria are now published (HHMI, 2019). As one element of the review, HHMI asks Investigators to "choose five of their most significant articles and provide a brief statement for each that describes the significance and impact of that contribution." It is worth noting that both published and preprint articles can be included. This emphasis on a handful of papers helps focus the review evaluation on the quality and impact of the Investigator's work.

Generic terms like 'world-class' or 'excellent' appear to provide standards for quality; however, they are so broad that they allow evaluators to apply their own definitions, creating room for bias.

Arguably, universities face a stiffer challenge here. Institutions striving to improve their research assessment practices will likely be casting anxious looks at what their competitors are up to. However, one of the hopeful lessons from the October meeting is that less courage should be required – and progress should be faster – if institutions come together to collaborate and establish a shared vision for the reform of research evaluation.

### Finding conceptual clarity

Conceptual clarity in hiring, promotion, and tenure policies is another area for institutions to examine when aligning practices with values (Hatch, 2019). Generic terms like 'world-class' or 'excellent' appear to provide standards for quality; however, they are so broad that they allow evaluators to apply their own definitions, creating room for bias. This is especially the case when, as is still likely, there is a lack of diversity in decision-making panels. The use of such descriptors can also perpetuate the Matthew Effect, a phenomenon in which resources accrue to those who are already well resourced. Moore et al., 2017 have critiqued the rhetoric of 'excellence' and propose instead focusing evaluation on more clearly defined concepts such as soundness and capacity-building. (See also Belcher and Palenberg, 2018 for a discussion of the many meanings of the words 'outputs', 'outcomes' and 'impacts' as applied to research in the field of international development).

### Establishing standards

Institutions should also consider conceptual clarity when structuring the information requested from those applying for jobs, promotion, or funding. There have been some interesting innovations in recent years from institutions seeking to advance more holistic forms of researcher evaluation. UMC Utrecht, the Royal Society, the Dutch Research Council (NWO), and the Swiss National Science Foundation (SNSF) are also experimenting with structured narrative CV formats (Benedictus et al., 2016; Gossink-Melenhorst, 2019; Royal Society, 2020; SNSF, 2020). These can be tailored to institutional needs and values. The concise but consistently formatted structuring of information in such CVs facilitates comparison between applicants and can provide a richer qualitative picture to complement more the quantitative aspects of academic contributions.

DORA worked with the Royal Society to collect feedback on its 'Resumé for Researchers' narrative CV format, where, for example, the author provides personal details (e.g., education, key qualification and relevant positions), a personal statement, plus answers to the following four questions: how have you contributed to the generation of knowledge?; how have you contributed to the development of individuals?; how have you contributed to the wider research community?; how have you contributed to broader society? (The template also asks about career breaks and other factors "that might have affected your progression as a researcher"). The answers to these questions will obviously depend on the experience of the applicant but, as Athene Donald of Cambridge University has written: "The topics are broad enough that most people will be able to find something to say about each of them. Undoubtedly there is still plenty of scope for the cocky to hype their life story, but if they can only answer the first [question], and give no account of mentoring, outreach or conference organization, or can't explain why what they are doing is making a contribution to their peers or society, then they probably aren't 'excellent' after all" (Donald, 2020).

It is too early to say if narrative CVs are having a significant impact, but according to the NWO their use has led to an increased consensus between external evaluators and to a more diverse group of researchers being selected for funding (DORA, 2020).

Even though the imposition of structure promotes consistency, there is a confounding factor of reviewer subjectivity. At the meeting, participants identified a two-step strategy to reduce the impact of individual subjectivity on decision-making. First, evaluators should identify and agree on specific assessment criteria for all the desired capabilities. The faculty in the biology department at University of Richmond, for example, discuss the types of expertise, experience, and characteristics desired for a role *before* soliciting applications.

This lays the groundwork for the second step, which is to define the full range of performance standards for criteria to be used in the evaluation process. An example is the three-point rubric used by the Office for Faculty Equity and Welfare at University of California, Berkeley, which helps faculty to judge the commitment of applicants to advancing diversity, equity, and inclusion (UC Berkeley, 2020). A strong applicant is one who "describes multiple activities in depth, with detailed information about both their role in the activities and the outcomes. Activities may span research, teaching and service, and could include applying their research skills or expertise to investigating diversity, equity and inclusion." A weaker candidate, on the other hand, is someone who provides "descriptions of activities that are brief, vague, or describe being involved only peripherally."

### Recognizing collaborative contributions

Researcher evaluation is rightly preoccupied with the achievements of individuals, but increasingly, individual researchers are working within teams and collaborations. The average number of authors per paper has been increasing steadily since 1950 (National Library of Medicine, 2020). Teamwork is essential to solve the most complex research and societal challenges, and is often mentioned as a core value in mission statements, but evaluating collaborative contributions and determining who did what remains challenging. In some disciplines, the order of authorship on a publication can signal how much an individual has contributed; but, as with other proxies, it is possible to end up relying more on assumptions than on information about actual contributions.

More robust approaches to the evaluation of team science are being introduced, with some aimed at behavior change. For example, the University of California Irvine has created guidance for researchers and evaluators on how team science should be described and assessed (UC Irvine, 2019). In a separate development, led by a coalition of funders and universities, the Contributor Roles Taxonomy (CRediT) system (https://credit.niso.org), which provides more granular insight into individual contributions to published papers, is being adopted by many journal publishers. But new technological solutions are also needed. For scientific papers, it is envisioned that authorship credit may eventually be assigned at a figure level to identify who designed, performed, and analyzed specific experiments for a study. Rapid Science is also experimenting with an indicator to measure effective collaboration (http://www.rapidscience.org/about/).

### Communicate the vision on campus and externally

Although many individual researchers feel constrained by an incentive system over which they have little control, at the institutional level and beyond they can be informed about and involved in the critical re-examination of research assessment. This is crucial if policy changes are to take root, and can happen in different ways, during and after the deliberations of the working groups described above. For example, University College London (UCL) held campus-wide and departmental-level consultations in drafting and reviewing new policies on the responsible use of bibliometrics, part of broader moves to embrace open scholarship (UCL, 2018; Ayris, 2020). The working group at Imperial College London organized a symposium to foster a larger conversation within and beyond the university about implementing its commitment to DORA (Imperial College, 2018).

Other institutions and departments have organized interactive workshops or invited speakers who advocate fresh thinking on research evaluation. UMC Utrecht, one of the most energetic reformers of research assessment, hosted a series of town hall meetings to collect faculty and staff input before formalizing its new policies. It is also working with social scientists from Leiden University to monitor how researchers at UMC are responding to the changes. Though the work is yet to be completed, they have identified three broad types of response: i) some researchers have embraced change and see the positive potential of aligning assessment criteria with real world impact and the diversity of academic responsibilities; ii) some would prefer to defend a status quo that re-affirms the value of more traditional metrics; iii) some are concerned about the uncertainty that attends the new norms for their assessment inside and outside UMC (Benedictus et al., 2019). This research serves to maintain a dialogue about change within the institution and will help to refine the content and implementation of research assessment practices. However, the changes have already empowered PhD students at UMC to reshape their own evaluation by bringing a new emphasis on research competencies and professional development to the assessment of their performance (Algra et al., 2020).

We encourage institutions and departments to publish information about their research assessment policies and practices so that research staff can see what is expected of them and, in turn, hold their institutions to account.

The Berlin Institute of Health (BIH) has executed a similarly deep dive into its research culture. In 2017, as part of efforts to improve its research and research assessment practices, it established the QUEST (Quality-Ethics-Open Science-Translation) Center in and launched a programme of work that combined communication, new incentives and new tools to foster institutional culture change (Strech et al., 2020). Moreover, a researcher applying for promotion at the Charité University Hospital, which is part of BIH, must answer questions about their contributions to science, reproducibility, open science, and team science, while applications for intramural funding are assessed on QUEST criteria that refer to robust research practices (such as strategies to reduce the risk of bias, and transparent reporting of methods and results). To help embed these practices independent QUEST officers attend hiring commissions and funding reviewers are required to give structured written feedback. Although the impact of these changes is still being evaluated, lessons already learned include the importance of creating a positive narrative centered on improving the value of BIH research and of combining strong leadership and tangible support with bottom-up engagement by researchers, clinicians, technicians, administrators, and students across the institute (Strech et al., 2020).

Regardless of format, transparency in the communication of policy and practice is critical. We encourage institutions and departments to publish information about their research assessment policies and practices so that research staff can see what is expected of them and, in turn, hold their institutions to account. While transparency increases accountability, it has been argued that it may stifle creativity, particularly if revised policies and criteria are perceived as overly prescriptive. Such risks can be mitigated by dialogue and consultation, and we would advise institutions to emphasize the spirit, rather than the letter, of any guidance they publish.

Universities should be encouraged to share new policies and practices with one another. Research assessment reform is an iterative process, and institutions can learn from the successes and failures of others. Workable solutions may well have to be accommodated within the traditions and idiosyncrasies of different institutions. DORA is curating a collection of new practices in research assessment that institutions can use as a resource (see sfdora.org/goodpractices), and is always interested to receive new submissions. Based on feedback from the meeting, one of us (AH) and Ruth Schmidt (Illinois Institute of Technology) have written a briefing note that helps researchers make the case for reform to their university leaders and helps institutions experiment with different ideas and approaches by pointing to five design principles for reform (Hatch and Schmidt, 2020).

## Looking ahead

DORA is by no means the only organization grappling with the knotty problem of reforming research evaluation. The Wellcome Trust and the INORMS research evaluation group have both recently released guidance to help universities develop new policies and practices (Wellcome, 2020b; INORMS, 2020). Such developments are aligned with the momentum of the open research movement and the greater recognition by the academy of the need to address long-standing inequities and lack of diversity. Even with new tools, aligning research assessment policies and practices to an institution's values is going to take time. There is tension between the urgency of the situation and the need to listen to and understand the concerns of the community as new policies and practices are developed. Institutions and individuals will need to dedicate time and resources to establishing and maintaining new policies and practices if academia is to succeed in its oft-stated mission of making the world a better place. DORA and its partners are committed to supporting the academic community throughout this process.

## Note

DORA receives financial support from eLife, and an eLife employee (Stuart King) is a member of the DORA steering committee.
